# Effects of a Short-Term “Fat Adaptation with Carbohydrate Restoration” Diet on Metabolic Responses and Exercise Performance in Well-Trained Runners

**DOI:** 10.3390/nu13031033

**Published:** 2021-03-23

**Authors:** Kaixuan Che, Junqiang Qiu, Longyan Yi, Menghui Zou, Zhihui Li, Amelia Carr, Rhiannon M.J. Snipe, Dan Benardot

**Affiliations:** 1 Department of Exercise Biochemistry, Exercise Science School, Beijing Sport University, Beijing 100084, China; 2019210241@bsu.edu.cn (K.C.); 2136@bsu.edu.cn (L.Y.); LiZhiHui0817@hotmail.com (Z.L.); 2 China Athletics School, Beijing Sport University, Beijing 100084, China; zoumh@bsu.edu.cn; 3 Centre for Sport Research, Faculty of Health, Deakin University, Melbourne, VIC 3125, Australia; amelia.carr@deakin.edu.au (A.C.); r.snipe@deakin.edu.au (R.M.J.S.); 4 Department of Nutrition, Georgia State University, Atlanta, GA 30303, USA; dan.benardot@emory.edu; 5 Center for the Study of Human Health, Emory University, Atlanta, GA 30322, USA

**Keywords:** periodized nutrition, high carbohydrate, high fat diet, glycogen restoration

## Abstract

Periodized carbohydrate availability can enhance exercise capacity, but the effects of short-term fat adaptation carbohydrate restoration (FACR) diets on metabolic responses and exercise performance in endurance athletes have not been conclusively determined. This study aimed to investigate the effect of a FACR diet on measures of resting metabolism, exercise metabolism, and exercise performance. Well-trained male runners (*n* = 8) completed a FACR dietary intervention (five days’ carbohydrate < 20% and fat > 60% energy, plus one-day carbohydrate ≥ 70% energy), and a control high-carbohydrate (HCHO) diet for six days (carbohydrate > 60% energy; fat < 20% energy) in a randomized crossover design. Pre- and post-intervention metabolic measures included resting metabolic rate (RMR), respiratory quotient (RQ), maximum fat oxidation rate during exercise (MFO), and maximum fat oxidation intensity (FATmax). Measures of exercise performance included maximal oxygen uptake (VO_2_max), running economy (RE), and 5 km running time trial (5 km-TT). In FACR compared with HCHO, there were significant improvements in FATmax (*p* = 0.006) and RE (*p* = 0.048). There were no significant differences (*p* > 0.05) between FACR and HCHO in RMR, RQ, VO_2_max, or 5 km-TT. Findings suggest that a short-term (six days) FACR diet may facilitate increased fat oxidation and submaximal exercise economy but does not improve 5 km-TT performance.

## 1. Introduction

A periodized carbohydrate intake strategy requires athletes to systematically incorporate periods of low carbohydrate intake (e.g., days or weeks), with high carbohydrate intake implemented at specified time points to support key training sessions [1]. This dietary strategy has in some cases been implemented by athletes [2,3], and may change substrate utilization (e.g., increase fat oxidation) [4], support endurance exercise training adaptations [5], and improve high-intensity exercise performance in sub-elite athletes [1]. One nutritional strategy that can be implemented to achieve a periodized carbohydrate intake is the administration of a non-ketogenic high-fat, low-carbohydrate diet (a restricted carbohydrate availability diet, which also retains sufficient carbohydrate to avoid sustained ketosis [6]) for a specified period of time for adapting to increased fat metabolism, followed by a short period of high carbohydrate intake (carbohydrate restoration; [6]).

Over the past twenty years, short-term fat-adaptation/carbohydrate restoration (FACR) diets have been investigated, and typically encompass at least five days of 15–20% energy intake from carbohydrate, and 60–65% energy from fat, followed by 24 h of a high-carbohydrate diet (10–12 g/kg per day; [6]). Reported effects of FACR interventions include an increased contribution of fat as a substrate for exercise performance, via increased rates of whole-body fat oxidation [5], and increases in intra-muscular triglyceride stores, fatty acid mobilization, and transport. Performance in shorter duration endurance running events, such as five km running efforts, after FACR diets have yet to be investigated.

High-fat, low-carbohydrate dietary interventions are typically challenging to implement within both research and practical contexts, due to the substantial changes required to modify typical dietary intakes, and the expense and limited availability of specific foods [7]. Recently, there has been an increased prevalence in the use of smartphone applications, which can assist with dietary prescription and monitoring nutritional intake [8,9]. Such innovations may enable enhanced practitioner dietary prescription and dietary evaluation for athletes to adhere to challenging dietary strategies, such as a FACR dietary intervention, especially if they choose to implement such dietary strategies at specific time points, such as prior to key sporting events or races.

It is currently unclear as to the effects of a six-day FACR dietary intervention on 5 km running, which is amongst the endurance running events with the highest global participation rates [10], and measures of fat and carbohydrate oxidation. Therefore, this study presents a preliminary investigation of a practical model for the implementation of a FACR dietary intervention, using recently developed technologies (mobile phone applications). The aim of this pilot study was to determine the effect of a short-term FACR diet as prescribed using a mobile phone application on measures of carbohydrate and fat oxidation, and physical performance capacity, in the context of a 5 km running time trial event.

## 2. Materials and Methods

### 2.1. Participants

Well-trained runners were recruited by advertising to marathon running clubs. A pre-screening questionnaire, which assessed medical, dietary, training, and running competition histories, was completed by well-trained runners to determine eligibility for inclusion in the study. Inclusion criteria comprised healthy male endurance runners aged 18–50 years, regular eating habits without following specific diet types (e.g., ketogenic diet or low carbohydrate diet), and national first-class or second-class athletes (finalists in provincial competitions within China) for running events >800 m. Eight well-trained runners met the inclusion criteria (Table 1) and included gold medalists in the 5000 m event (time 15 min, 40 s) of the 14th Beijing Games in 2014; the gold medalist in the 800 m event (personal best time 1 min 53 s) and 1500 m event (personal best time 4 min 4 s) in the 2014–2016 Hebei Elite Athletics Championship, and/or top 10% finish in a sanctioned marathon (e.g., a gold medalist of the 2018 Xuzhou International Half Marathon, with a score of 1 h, 8 min, 6 s).

The research proposal was conducted in compliance with the declaration of Helsinki and approved by the Institutional Review Board of Beijing Sport University (BSU IRB). All participants gave written informed consent prior to study participation (No. 2018025H).

### 2.2. Experimental Testing Overview

**Figure 1:**
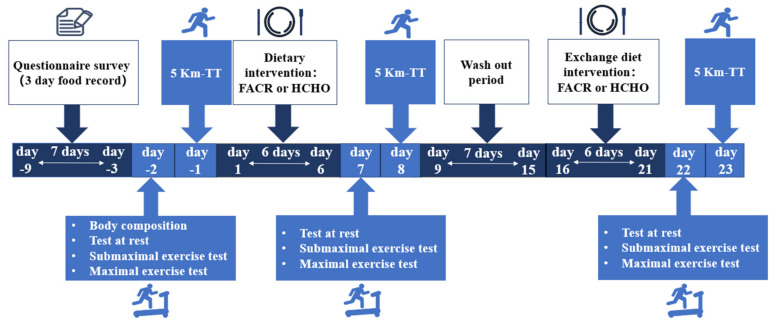
Experimental testing overview. The test at rest and exercise test were performed three times, that is, before any intervention, then after fat adaptation carbohydrate restoration (FACR) (or control high-carbohydrate (HCHO)), and then after HCHO (or FACR). Resting metabolic rate (RMR) and respiratory quotient (RQ) were measured in the test at rest. Maximum fat oxidation rate (MFO), maximum fat oxidation intensity (FATmax), oxygen consumption (VO_2_) under FATmax (VO_2_ (FATmax)), running speed under FATmax (Speed (FATmax)), running economy (RE), heart rate (HR), VO_2_max and 5 km time trial (5-km TT) were measured in exercise tests.

Baseline measures, consisting of a three-day food record and body composition assessment (Inbody 720, Biospace Co., Ltd., Seoul, Korea), were completed at baseline prior to experimental testing. Participants then completed two dietary interventions that included an experimental FACR diet and a control high carbohydrate (HCHO) diet in a randomized crossover design with a seven-day washout period (Figure 1). Testing was completed over two days at baseline and following each dietary intervention. Day one of testing included measures of resting metabolic rate (RMR; kcal) and respiratory quotient (RQ; VCO_2_/VO_2_) followed by a six-minute sub-maximum exercise test at a treadmill speed of 11.5 km·h^−1^ to determine running economy (RE; mL·kg·min^−1^), which is quantified as the O_2_ cost for a given velocity, estimated from measuring steady-state O_2_ consumption (VO_2_) during submaximal running [11]. It has been established that runners with a higher RE will record a lower VO_2_ at a given steady-state speed than runners with a lower RE [12]. In addition, a maximal exercise test was performed to determine maximal oxygen consumption (VO_2_max; mL·kg·min^−1^) and maximum fat oxidation (MFO; g·min^−1^). On day two of testing, participants completed a 5 km running time trial (5 km-TT) test (Figure 1).

### 2.3. Experimental Protocol

#### 2.3.1. Diet Plan

Participants completed a three-day food record of their habitual diet over two weekdays and one weekend day [13] to determine baseline nutrient (% energy intake from carbohydrate, fat, and protein) intake one week prior to the experimental intervention. Participants were familiarized with the nutrient analysis application (Boohee, Information Technology Co., Ltd., Shanghai, China) [14,15] that was installed on the participant’s smartphone and used to complete the three-day food record. Portion sizes for all consumed foods and liquids were estimated using standard-sized bowls and dishes as the unit, with the nutrient analysis provided by the application.

The application was used to develop personalized recipe suggestions for each participant, based on their habitual intake and prescribed dietary interventions, which consisted of (1) a five-day non-ketogenic, high-fat (>60% energy intake) low-carbohydrate (<20% energy intake) diet followed by a one-day high-carbohydrate (≥70% energy intake) diet to restore muscle glycogen (FACR) [6]; and (2) a high-carbohydrate (carbohydrate > 60% energy intake; fat < 20% energy intake) diet for six days (HCHO) in a randomized crossover design with a seven-day washout period [16,17]. Participant adherence to their dietary plan was monitored using the application’s diet tracking function that validated intake from photos of food and fluids that were taken and uploaded to the application by each participant. Upon completion of the study, energy intake distributions (%) from carbohydrate, protein, and fat were downloaded from the application and recorded (Table 2).

#### 2.3.2. Resting Metabolism Test

During the data collection period, participants performed their normal training at least twice per week, and refrained from any competition for a minimum of seven days prior to testing and were required to rest for 12 h prior to testing. Participants were required to abstain from alcohol or caffeine for 48 h prior to each two-day testing period to avoid any potential effects on exercise performance or heart rate. Participants arrived at the laboratory after a 10-h overnight fasting period and sat in a seated posture for 20 min upon arrival. Resting energy expenditure was quantified via indirect calorimetry (COSMED Quark PFT Ergo, Roma, Italy). Resting metabolic rate (RMR, kcal·d^−1^) testing was then conducted, using a metabolic cart (COSMED Quark PFT Ergo, Roma, Italy). The RQ (VCO_2_/VO_2_) was recorded and RMR (kcal·d^−1^) was calculated by the Weir formula [18].

#### 2.3.3. Submaximal Exercise Protocol

Upon completion of the resting metabolism test, participants performed a submaximal running test on a motorized treadmill (h/p/cosmos pulsar, Munich, Germany) to determine running economy (RE). The test consisted of a three-minute warm-up, at a speed of five km·h^−1^ and a gradient of zero followed by six minutes running at a treadmill speed of 11.5 km·h^−1^. Oxygen consumption (mL·kg·min^−1^) was collected for a three-minute period once steady-state VO_2_ was reached, and this value was recorded as the RE [19]. Heart rate (HR, bpm) was measured continuously during this period and the mean value during the three-minute steady state was recorded (Polar V800, Polar, NY, USA).

#### 2.3.4. Maximal Aerobic Capacity

Maximal oxygen uptake (VO_2_max) testing was conducted on a motorized treadmill following completion of the submaximal running test, and a 30-min rest period. The protocol began with a three-minute walking warm-up at 5 km·h^−1^. The starting speed for the test was 6.8 km·h^−1^, followed by three-minute incremental stages with a 1.2 km·h^−1^ increase in speed at each stage, and the gradient remained unchanged at zero throughout the process. The protocol used was similar to previously reported protocols [20,21]. The criteria for attaining VO_2_max was achieving at least three of the following criteria: (1) VO_2_ did not increase with the increase of load, and the participants voluntarily stopped the treadmill when they were exhausted; (2) the variation range of VO_2_ did not exceed 5% or 150 mL·min^−1^ or 2 mL·kg·min^−1^; (3) RQ > 1.1; and (4) HR > 180 bpm.

During the test, ventilation samples were collected continuously using an indirect calorimetry system, (COSMED Quark PFT Ergo, Roma, Italy), which was calibrated prior to each test. VO_2_ and VCO_2_ were recorded every 30 s and used to calculate fat oxidation rates [22]. Maximum fat oxidation rate (MFO, g·min^−1^) per unit fat mass was determined as the highest recorded value of the last 30 s interval of each stage, and the corresponding intensity was the maximum fat oxidation exercise intensity (FATmax, %VO_2_max). MFO during exercise has been widely reported and can reflect the ability to maximize fat utilization [23]. In addition, VO_2_ (mL·kg·min^−1^) and running speed (km·h^−1^) at the same intensity were recorded. Heart rate (Polar V800, Polar, NY, USA) was measured throughout the test.

#### 2.3.5. Five km Time Trial

Participants followed the diet consistent with their last day of treatment (FACR or HCHO) within 24 h before the 5 km-TT (e.g., high in carbohydrate under both conditions) to restore muscle glycogen and included a high-carbohydrate breakfast. On day two of testing, participants returned to the laboratory. After a 20-min rest period, participants completed a five-minute warm-up at a self-selected speed. Participants then performed an individual 5 km running time trial on an outdoor synthetic track-and-field ground, and the time was recorded using a stopwatch (CASIO HS-70W, Tokyo, Japan).

### 2.4. Statistical Analyses

Statistical analyses were undertaken using SPSS 17.0. For baseline data (Table S1), a paired samples *t*-tests were used to compare test data (RMR, RQ, MFO, FATmax, VO_2_ (FATmax), Speed (FATmax), RE, HR(RE), VO_2_max, 5 km-TT) between baseline and the dietary interventions (FACR or HCHO). Data from the two dietary conditions (Tables S2 and S3) were compared using a two-factor (diet and time) analysis of variance (ANOVA) with repeated measures. Tukey–Kramer post hoc tests were undertaken when ANOVA revealed a significant difference or interaction effects were observed. Significance was accepted at *p <* 0.05. All data are reported as $\bar{x\;}$ ± SD (Table S4).

## 3. Results

### 3.1. MFO and FATmax

**Figure 2:**
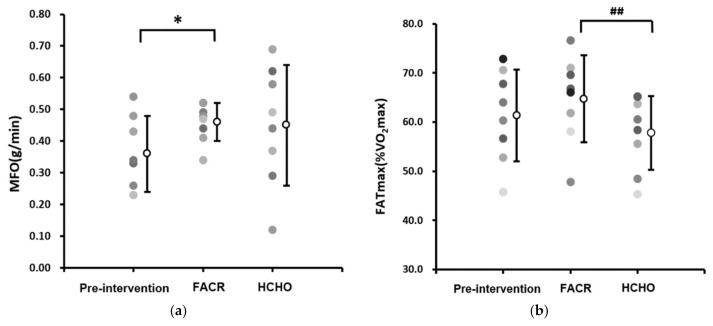
Individual (**a**) maximum fat oxidation rate (MFO) and (**b**) maximum fat oxidation intensity (FATmax) during a maximal graded treadmill test pre-intervention and after fat adaptation/carbohydrate restoration (FACR) and high carbohydrate (HCHO) diets. * Significantly different to pre-intervention (*p* < 0.05). ^##^ Significantly different to HCHO control (*p* < 0.01).

There was a significant increase in MFO from pre to post-intervention in FACR (0.36 ± 0.12 g·min^−1^ vs. 0.46 ± 0.06 g·min^−1^, *p* = 0.037), but no significant difference was observed in HCHO (0.36 ± 0.12 g·min^−1^ vs. 0.45 ± 0.19 g·min^−1^, *p* = 0.258) or between FACR and HCHO (0.46 ± 0.06 g·min^−1^ vs. 0.45 ± 0.19 g·min^−1^, *p* = 0.919) (Figure 2a). There was no significant increase in FATmax from pre to post-intervention in FACR (61.27 ± 9.26% VO_2_max vs. 64.74 ± 8.87% VO_2_max, *p* = 0.341) and HCHO (61.27 ± 9.26% VO_2_max vs. 57.79 ± 7.57% VO_2_max, *p* = 0.171). However, FATmax was significantly higher for FACR compared with HCHO (64.74 ± 8.87% VO_2_max vs. 57.79 ± 7.57% VO_2_max, *p* = 0.006) (Figure 2b).

### 3.2. Resting Metabolism

**Figure 3:**
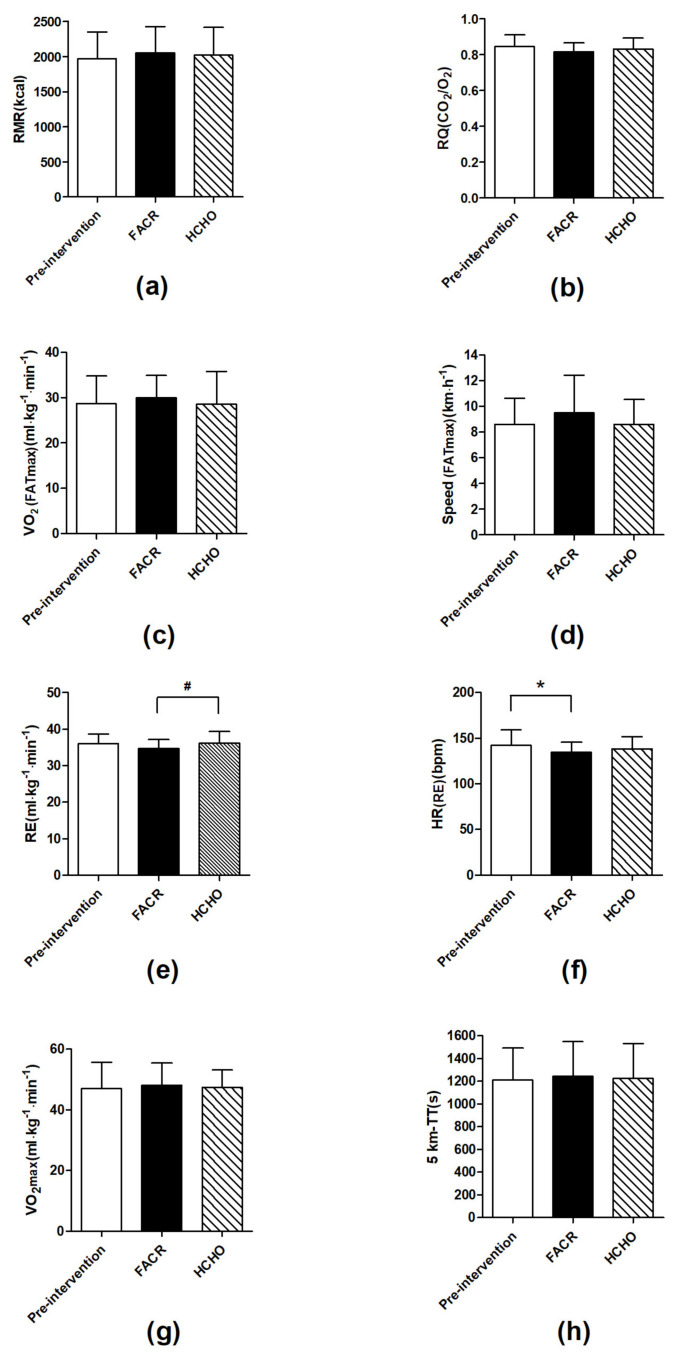
(**a**) Resting metabolic rate (RMR), (**b**) respiratory quotient (RQ), (**c**) VO_2_ under FATmax (VO_2_ (FATmax)), (**d**) running speed under FATmax (Speed (FATmax)), (**e**)running economy (RE), (**f**) heart rate under RE (HR(RE)), (**g**) VO_2_max and (**h**) 5 km time trial (5-km TT) pre-intervention and after fat adaptation/carbohydrate restoration (FACR) and high carbohydrate (HCHO) diets. * Significantly different to pre-intervention (*p* < 0.05)^. #^ Significantly different to HCHO control (*p* < 0.05).

There was no significant difference in RMR from pre to post-intervention in FACR (1968.19 ± 383.68 kcal·d^−1^vs. 2051.21 ± 375.68 kcal·d^−1^, *p* = 0.625) and HCHO (1968.19 ± 383.68 kcal·d^−1^ vs. 2017.81 ± 397.00 kcal·d^−1^, *p* = 0.797) or between FACR and HCHO (2051.21 ± 375.68 kcal·d^−1^ vs. 2017.81 ± 397.00 kcal·d^−1^, *p* = 0.707) (Figure 3a). There was no significant difference in RQ from pre to post-intervention in FACR (0.84 ± 0.07 vs. 0.82 ± 0.05, *p* = 0.430) and HCHO (0.84 ± 0.07 vs. 0.83 ± 0.06, *p* = 0.613) (Figure 3b) or between FACR and HCHO (0.82 ± 0.05 vs. 0.83 ± 0.06, *p* = 0.647).

### 3.3. VO_2_ (FATmax) and Speed (FATmax)

There was no significant difference in VO_2_ (FATmax) from pre to post-intervention in FACR (28.73 ± 6.05 mL·kg·min^−1^ vs. 29.94 ± 4.98 mL·kg·min^−1^, *p* = 0.197) and HCHO (28.73 ± 6.05 mL·kg·min^−1^ vs. 28.55 ± 7.23 mL·kg·min^−1^, *p* = 0.220) or between FACR and HCHO (29.94 ± 4.98 mL·kg·min^−1^ vs. 28.55 ± 7.23 mL·kg·min^−1^, *p* = 0.479) (Figure 3c). There was no significant difference in speed (FATmax) from pre to post-intervention change in FACR (8.60 ± 2.03 km/h vs. 9.50 ± 2.92 km/h, *p* = 0.285) and HCHO (8.60 ± 2.03 km/h vs. 8.60 ± 1.92 km/h, *p* = 1.000) or between FACR and HCHO (9.50 ± 2.92 km/h vs. 8.60 ± 1.92 km/h, *p* = 0.176) (Figure 3d).

### 3.4. RE, HR(RE), VO_2_max and Five km-TT

There was no significant difference in RE (running economy) from pre to post-intervention in FACR (36.07 ± 2.65 mL·kg·min^−1^ vs. 34.64 ± 2.54 mL·kg·min^−1^, *p* = 0.097) and HCHO (36.07 ± 2.65 mL/kg·min^−1^ vs. 36.11 ± 3.26 mG·kg·min^−1^, *p* = 0.967). However, RE (mL·kg·min^−1^) was significantly lower in FACR compared with HCHO (34.64 ± 2.54 mL·kg·min^−1^ vs. 36.11 ± 3.26 mL·kg·min^−1^, *p* = 0.048) (Figure 3e). There was a significant decrease in HR(RE) from pre to post-intervention in FACR (142.13 ± 16.75 bpm vs. 134.38 ± 11.01 bpm, *p* = 0.048); but not in HCHO (142.13 ± 16.75 bpm vs. 138.25 ± 13.54 bpm, *p* = 0.566). HR(RE) in FACR was not significantly different to HCHO (134.38 ± 11.01 bpm vs. 138.25 ± 13.54 bpm, *p* = 0.440) (Figure 3f).

There was no significant difference in VO_2_max from pre to post-intervention in FACR (47.08 ± 8.59 mL·kg·min^−1^ vs. 48.12 ± 7.30 mL·kg·min^−1^, *p* = 0.491) and HCHO (47.08 ± 8.59 mL·kg·min^−1^ vs. 47.36 ± 5.78 mL·kg·min^−1^, *p* = 0.847) or between FACR and HCHO (48.12 ± 7.30 mL·kg·min^−1^ vs. 47.36 ± 5.78 mL·kg·min^−1^, *p* = 0.428) (Figure 3g). There was no significant difference in 5 km -TT from pre to post-intervention in FACR (1210.38 ± 281.98 s vs. 1241.38 ± 306.45 s, *p* = 0.116) and HCHO (1210.38 ± 281.98 s vs. 1223.00 ± 307.88 s, *p* = 0.662) or between FACR and HCHO (1241.38 ± 306.45 s vs. 1223.00 ± 307.88 s, *p* = 0.318) (Figure 3h).

## 4. Discussion

In this pilot study, we investigated a practical model for implementing a short-term fat adaptation followed by carbohydrate restoration (FACR) diet in well-trained runners. Consistent with previous studies in the area, we also investigated markers of carbohydrate and fat metabolism and exercise performance. Key findings were a significant increase in the exercise intensity at which maximal fat oxidation rates occurred (FATmax), a significant increase in running economy, in FACR compared with a high carbohydrate (HCHO) control, and a significant increase in maximal fat oxidation rates from pre to post intervention in the FACR condition. No significant changes were detected in markers of carbohydrate metabolism, or 5 km time trial performance.

In the current investigation, maximal fat oxidation rates were achieved at a higher exercise intensity in FACR than with HCHO control, evidenced by a significantly higher FATmax after FACR. Given that under normal dietary conditions there is typically a decrease in fat oxidation rates as exercise intensity increases, the results of the current pilot study provide a preliminary indication that there may be differences in substrate selection [20,24] following the FACR intervention. Further studies on the effects of a FACR intervention on FATmax are required, however, given the small sample size used in the current study. Previous studies investigating FACR have not quantified FATmax but have investigated fat oxidation rates. In previous studies with similar, non-ketogenic FACR protocols to the current study, increased rates of fat oxidation and reduced carbohydrate oxidation rates have been observed during submaximal cycling [4,25,26].

In the current investigation, no significant differences were observed between FACR and control in maximal fat oxidation rates. The use of noninvasive methods of quantifying fat oxidation in the current pilot investigation may provide direction for future studies, which may use larger sample sizes and more direct methods. However, there was a significant difference in maximal fat oxidation rates in the FACR condition, between the pre-and post-intervention time points. The trend toward increased fat metabolism and reduced carbohydrate oxidation in endurance athletes after FACR, as indicated by the preliminary results provided by the current pilot study, and in previous studies [4,26,27,28,29] is suggestive of diet-induced adaptations that could enhance the training-induced increased capacity for fat adaptation observed in endurance-trained athletes [5].

Running economy in the current investigation was significantly improved in the FACR compared with the HCHO condition. This is the first study to investigate the effects of a FACR intervention on running economy, however, several previous studies have investigated similar measures of exercise economy and efficiency after high-fat dietary interventions [19,29,30]. In contrast to the findings of the current investigation, a recent study investigating elite race walkers after a short-term ketogenic diet (defined as severe carbohydrate restriction, and induced chronic ketosis [6]), and carbohydrate restoration period reported impaired exercise economy [29]. Similarly, submaximal running efficiency was impaired after a 31-day ketogenic diet intervention in trained endurance athletes [31]. In both previous studies, the ketogenic diet intervention (in contrast with the non-ketogenic high fat diet used in the current investigation) and increased reliance on fat oxidation may partially explain the increased oxygen cost when exercising at specific exercise intensities, given the substantially reduced carbohydrate availability associated with the substantially restricted carbohydrate intake typical of ketogenic diets (<50 g/day; [6]). The results presented within this study suggest that a non-ketogenic, short-term FACR diet may elicit a beneficial effect on running economy. While running economy is a valid predictor of running performance in runners [19], further investigation is required to address the limitation of small sample size within the current pilot study. It is also important to note that the current investigation found that running performance was maintained rather than improved in comparison to HCHO.

A unique feature of the current investigation was the quantification of performance in a 5 km time trial, in a setting that replicated real-life racing conditions. There were no significant differences between the FACR and HCHO conditions, and, therefore, no detrimental effect on performance after the FACR intervention. No previous study has quantified running performance after FACR, but previous investigations have reported impaired high-intensity exercise capacity, including a reduced power output in a 1 km cycling time trial after FACR compared with control [32]. The potential for impaired performances in high intensity exercise after FACR may be partially attributable to blunted pyruvate dehydrogenase activation and reduced carbohydrate oxidation [26], given that carbohydrate is the predominant fuel required for high-intensity exercise [30]. Further investigation of the effects on 5 km running performance is warranted, given that there was some variation in training status within our participant population, as indicted by the standard deviation in VO_2_max values.

Other observed responses to high fat diet interventions, such as reduced body mass and improved body composition [33], are also associated with improvements in high intensity running performance [34], which could also contribute to longer term performance outcomes for 5 km runners. Further investigation of the effect of repeated exposures to FACR over extended periods as a strategy to achieve low carbohydrate availability is warranted, given the prevalence of low carbohydrate availability dietary strategies in athletes [2,3] and reported performance benefits after systematically implemented low carbohydrate availability interventions [1].

The FACR protocol used in the current investigation may have practical benefits for trained runners. A six-day FACR intervention (five-day high fat diet) is likely to be more realistic and feasible to implement than longer term high fat dietary interventions [25]. Further, the use of mobile phone applications, as used in the current investigation, may further assist with the implementation of FACR. Given that a high fat intake requires substantial changes to typical dietary patterns, which for most athletes typically include a high proportion of carbohydrates [35], dietary monitoring is likely to be required, and the increased prevalence of mobile phone applications for dietary standardization [36] may reduce the burden on athletes when documenting their intake [37]. Applications are also typically easier to include within daily training routines than more traditional methods of recording dietary intake [36].

There are some limitations within the current investigation, which provide direction for future research in this area. Future studies should aim to include more direct measures of fat oxidation rates, and the quantification of additional, minimally invasive physiological measures (e.g., blood lactate concentration) using a similar study design would provide further insight into carbohydrate metabolism during exercise (e.g., maximal 5 km efforts). Future studies investigating FACR and running performance are required to build upon the findings of the current pilot study. Significantly lower mean plasma lactate concentration was previously observed during high intensity cycling training after FACR compared with control [32], and similar measures during a running-based study would facilitate comparisons between exercise modalities after FACR. In addition, no measures of ketones (acetoacetate, beta-hydroxybutyrate, and/or acetone) were undertaken on either the FACR or HCHO groups to determine dietary influence on ketone production. Rating of perceived exertion (RPE) is another noninvasive method that would provide additional detail to athletes’ responses to FACR interventions and high-intensity running, given that cycling-based studies have reported both consistent RPE for FACR and control conditions, coupled with impaired high-intensity performance, and increased RPE after FACR compared to HCHO at a fixed exercise intensity [32,38]. Additional subjective measures, which would more comprehensively document runners’ responses to FACR in competitive context, include the quantification of side effects, given that an increased severity and/or prevalence of gastrointestinal symptoms is a well-documented response to changes in carbohydrate intake [39,40], and fatigue-related symptoms, which have been reported after FACR in cyclists [25]. The standardization and quantification of training performed prior to and during the FACR intervention would allow further interpretation of the effectiveness of FACR interventions, particularly in the context of training responses quantified after several days of low carbohydrate intake. In the current investigation, there were significant differences in macronutrient intakes between the two dietary conditions, however, the mean carbohydrate intake during FACR was slightly higher than the recommended intake for FACR (≤20% of total energy intake; [6] and fat intake was slightly lower than recommended (>60%; [6]), which is a limitation of the current investigation. A unique element of the current investigation was the use of mobile phone applications to implement the dietary intervention. Potentially, this aspect of the methodology may have impacted participants’ ability to reduce their carbohydrate intake and increase fat intake. In future investigations, the use of smartphone applications could be supported by additional education provided to runners by accredited sports dietitians, given that applications are currently used by some dietitians, and the support provided when using applications can improve nutrition knowledge in athletes [41].

## 5. Conclusions

The results of this pilot study indicate that a short-term, non-ketogenic, high fat dietary intervention, followed by 24 h of carbohydrate restoration (FACR) can enhance measures of fat oxidation and running economy. Running performance after FACR did not differ from that observed in the control condition. However, further investigation is required to explore if 5 km running performance can be improved with repeated exposures to FACR over extended periods of time.

## Figures and Tables

**Table 1:** Participant characteristics, expressed as $\bar{x}$ (mean) and SD (standard deviation); male endurance athletes; *n* = 8.

	$\left(\bar{x}\pm \mathrm{SD}\right)$
Age (year)	27 ± 13
Height (cm)	177.1 ± 5.3
Body mass (kg)	65.6 ± 6.3
BMI (kg/m^2^)	20.9 ± 1.8
Body fat (%)	13.1 ± 7.1
VO_2_max(ml·kg·min^−1^)	47.08 ± 8.59

BMI: body mass index; VO_2_max: maximal oxygen uptake.

**Table 2:** Carbohydrate, fat and protein intake (% energy intake), for fat adaptation/carbohydrate restoration (FACR) and high carbohydrate (HCHO) dietary records ($\bar{x}$ ± SD).

	Pre-Intervention	Post-Intervention (FACR)		Post-Intervention (HCHO)
	1–3 d	1–5 d	6th d	1–6 d
CHO (%)	52.3 ± 5.8	23.4 ± 4.9 **^##^	66.3 ± 3.8 **^##^	59.4 ± 1.8 **
FAT (%)	31.5 ± 4.6	58.8 ± 4.2 **^##^	19.1 ± 4.4 **^##^	25.2 ± 2.1 **
PRO (%)	16.2 ± 2.0	17.9 ± 1.7 *^##^	14.6 ± 1.9 ^##^	15.4 ± 2.3 **

CHO, carbohydrate; PRO, protein. * Significantly different from pre-intervention (*p* < 0.05). ** Significantly different from pre-intervention (*p* < 0.01). ^##^ Significantly different from HCHO (*p* < 0.01).

## Data Availability

The following are available online at http://file.for.work/2021-1-31.pdf (accessed on 31 January 2021).

## Supplementary Materials

The following are available online at https://www.mdpi.com/2072-6643/13/3/1033/s1, Table S1: Individual RMR: RQ, MFO, FATmax, VO_2_ (FATmax), Speed (FATmax), RE, HR(RE), VO_2_max and 5-km TT of eight participants for pre-intervention. Table S2: Individual RMR, RQ, MFO, FATmax, VO_2_ (FATmax), Speed (FATmax), RE, HR(RE), VO_2_max and 5-km TT of eight participants for FACR. Table S3: Individual RMR, RQ, MFO, FATmax, VO_2_ (FATmax), Speed (FATmax), RE, HR(RE), VO_2_max and 5-km TT of eight participants for HCHO. Table S4: Comparing results of RMR, RQ, MFO, FATmax, VO_2_ (FATmax), Speed (FATmax), RE, HR(RE), VO_2_max and 5-km TT for pre and post-intervention (FACR or HCHO).
