# Supplementary figures and images for: Effects of a Short-Term “Fat Adaptation with Carbohydrate Restoration” Diet on Metabolic Responses and Exercise Performance in Well-Trained Runners
Source: Nutrients. 2021 Mar 23;13(3):1033. doi: 10.3390/nu13031033 (PMC8005046; doi:10.3390/nu13031033)

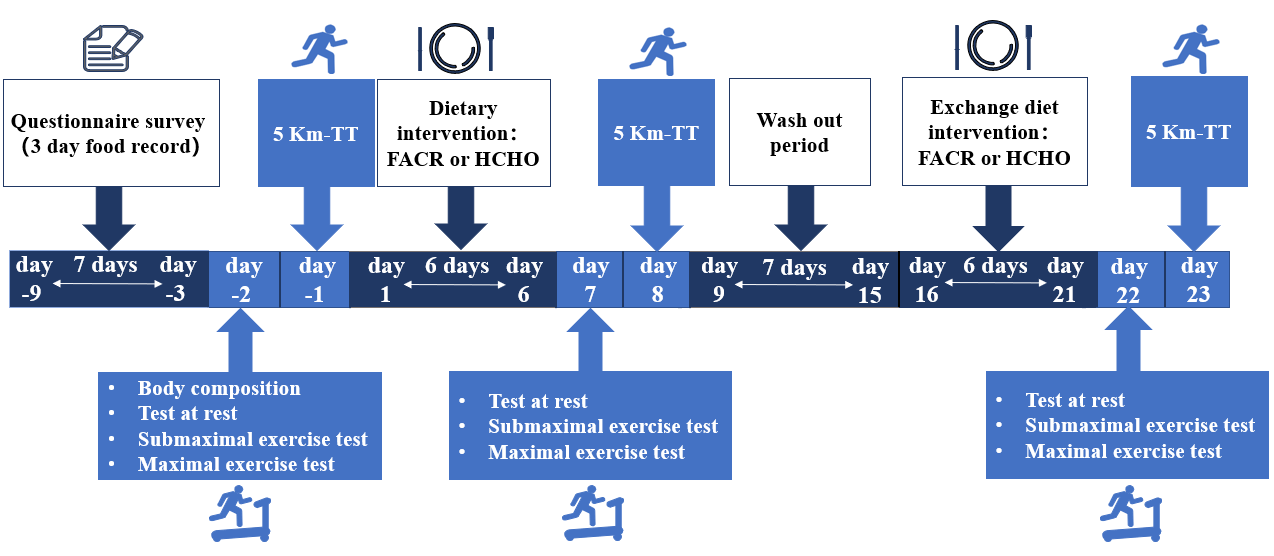

Supplement: Supplementary file 1 [file nutrients-13-01033-s001.zip › Figure 1.png]

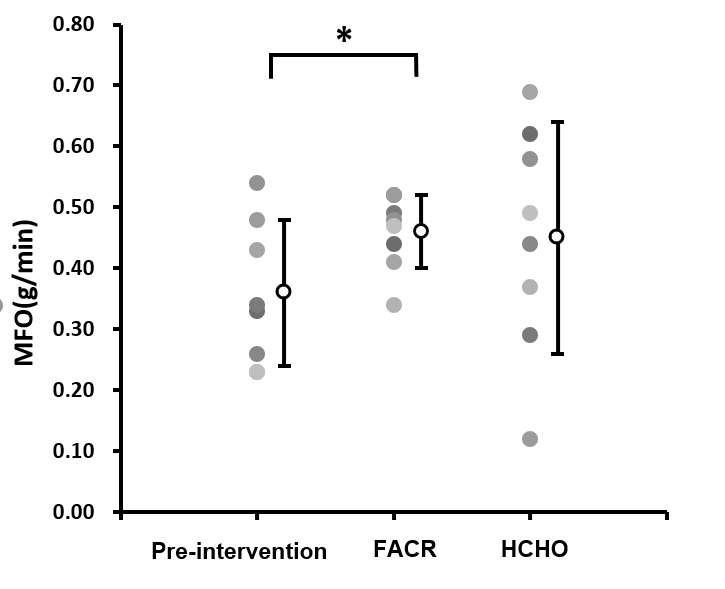

Supplement: Supplementary file 1 [file nutrients-13-01033-s001.zip › Figure 2a.png]

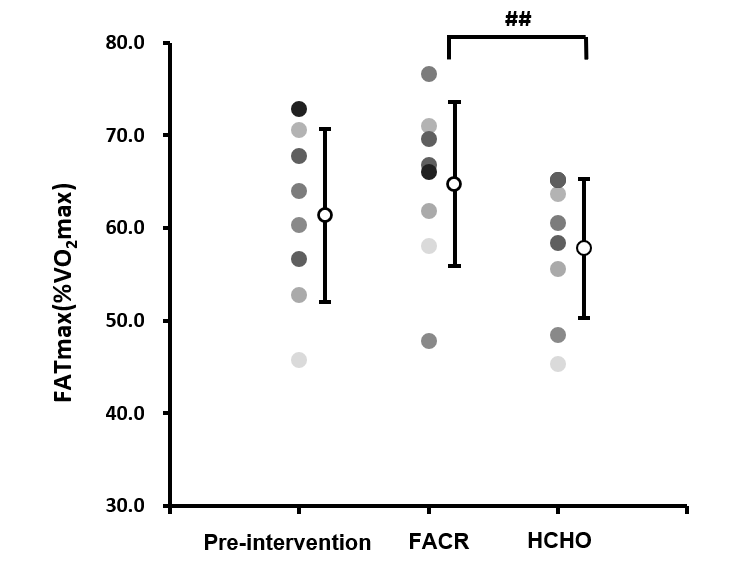

Supplement: Supplementary file 1 [file nutrients-13-01033-s001.zip › Figure 2b.png]

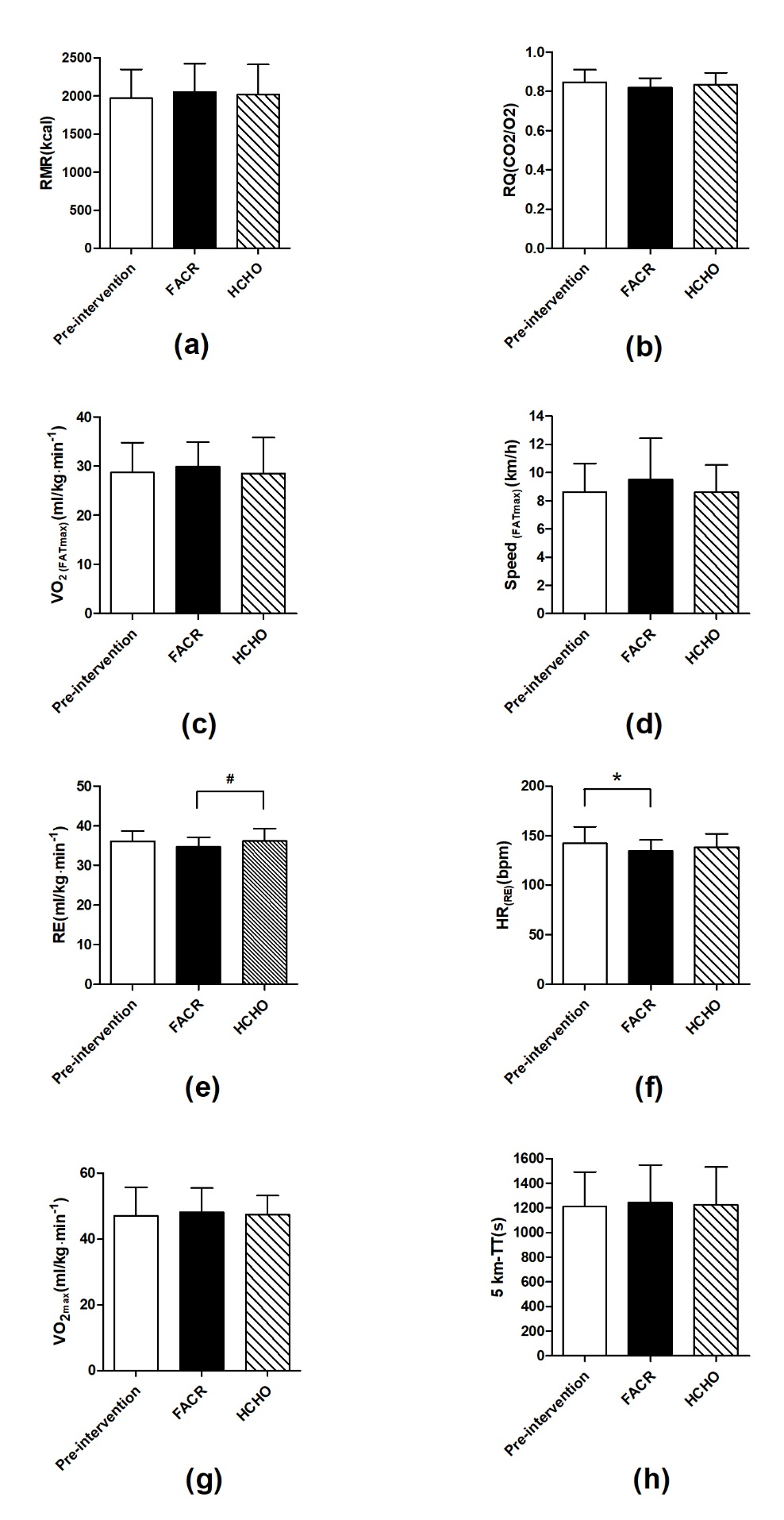

Supplement: Supplementary file 1 [file nutrients-13-01033-s001.zip › Figure 3.png]
